# Byte-sized progress: Will the current economic crisis in Sri Lanka impede the advancement of digital health citizenship in the country?

**DOI:** 10.1371/journal.pdig.0000843

**Published:** 2025-04-29

**Authors:** Udani De Silva, Mithara Fonseka, Sanduni Kotinkaduwa

**Affiliations:** 1 School of Public Health Sciences, Faculty of Health, University of Waterloo, Waterloo, Canada; 2 School of Environment, Enterprise and Development, Faculty of Environment, University of Waterloo, Waterloo, Canada; 3 Queen Elizabeth Hospital, National Health Service, London, United Kingdom; 4 School of Health and Medical Sciences, City St. George’s, University of London, London, United Kingdom; McGill University, CANADA

Since the wake of the COVID-19 pandemic, the growing interest in and demand for digital health heightened across public health systems and research across the world. Scientists from the Department of Civil and Systems Engineering at Johns Hopkins University had devised an https://www.arcgis.com/apps/dashboards/bda7594740fd40299423467b48e9ecf6 to record and visualize cases of COVID-19 in real-time globally. This web-based platform was first shared publicly on 22^nd^ January 2020 within a mere few weeks after the first case of COVID-19 had been identified in Wuhan, China and is still publicly accessible. However, such digital health initiatives seem to be a farfetched reality for most low- and middle-income countries (LMICs), including the island nation of Sri Lanka, where digital health has only begun to pick up momentum.

According to the World Bank, Sri Lanka is currently classified as an LMIC. Like many LMICs, the country’s healthcare administration is plagued by the various factors undermining digital health [1,2]. The general understanding is that developing countries have sparse resources and infrastructure to invest in technologies, including digital health [2,3]. Additionally, there is often a shortage of skilled technical experts for the establishment and maintenance of digital health systems. Considering that digital health technologies propagate at accelerated rates, the existing platforms are at a constant risk of redundancy before even being fully integrated into healthcare systems [2–4]. As a result, digital health administration in LMICs like Sri Lanka struggle to be on par with the ever-evolving landscapes of digital health. These gaps in healthcare standardization between high-income countries versus LMICs form obvious inequities in digital health, coined the “digital divide”.

Despite these challenges, Sri Lanka has made a significant stride towards accessible and equitable digital health in the country. The 2023 Performance and Progress Report by Sri Lanka’s Ministry of Health [5] describes key digital initiatives launched by the government. Sri Lanka has been certified as malaria-free by the World Health Organization since 2016, and the Anti-Malaria Campaign focuses on malaria surveillance and awareness, especially among travelers [5]. The provision of anti-malarial chemoprophylaxis adopts an online platform, and follow-up of suspected cases relies on online systems and WhatsApp groups. The National STD/AIDS Control Programme uses an electronic information management system to document electronic patient records, functioning across 28 clinics countrywide [5,6]. The Programme also refurbished a website dedicated to HIV testing called know4sure.lk [7], operating in the country’s three official languages, providing persons at risk with confidential support and self-testing kits.

The resilience of the Sri Lankan healthcare system was evident over the past several years as the country’s economy underwent a massive downturn [8]. Sri Lanka experienced an economic and political implosion in 2022 following years of structural inefficiencies and macroeconomic mismanagement, combined with a very recent history of exogenous shocks (i.e., COVID-19, 2019 Easter attacks, global oil price hikes) that further exacerbated an already vulnerable economy [8]. Consequently, the acute shortages in essential items like fuel, food, and medicines profoundly impacted public health and healthcare [8,9]. For instance, the threat of malnutrition loomed over young children due to crisis-induced food insecurity. To monitor children aged below 5 years with severe acute malnutrition, a mobile application was developed for public health midwives to follow children’s health and enter data into the application [5,8,9].

These digital health platforms exemplify the resilience of Sri Lankan healthcare systems during dire health crises. However, they also grapple with important challenges at present.

## 1. Sustainment of platforms

The Government of Sri Lanka devised several initiatives to tackle the burden of COVID-19, forming the national COVID-19 surveillance system of Sri Lanka. Through the COVID-19 Immunization Tracker, the Ministry of Health produced open-source, real-time data on vaccination, enabling the issuance of verifiable, digitized proof of COVID-19 vaccination, called a Smart Vaccination Certificate (SVC) [10]. However, the once efficient and fully functional SVC system has since been suspended, citing technical issues. Similarly, disease surveillance dashboards cannot be retrieved via Google searches, making health data inaccessible and not public knowledge.

## 2. Brain drain

While the emigration of trained professionals has not been a recent issue in Sri Lanka, the continuous loss of young professionals and experts threatens digital health citizenship like every other industry [11,12]. An estimated 297,656 Sri Lankans emigrated for foreign employment in 2023, for reasons including lucrative employment, better livelihoods, and improved working environments [13]. The abundance of employment opportunities, higher salaries, better living conditions, and economic and political stability are consistently known to be the push and pull factors behind the great exodus [13]. While reliable statistics are unavailable, many local news outlets report on the mass emigration of medical professionals and software developers in recent years, and that the issue is both one of quantity and quality, with specialists being high among the numbers who emigrate [11].

## 3. A divide within a divide

Another continual challenge infesting Sri Lankan healthcare systems is the inequity in access to not only digital health but healthcare, medication, and technological resources—A divide within a divide. Health research in Sri Lanka has consistently unearthed inequities in healthcare access and disease outcomes by geographical regions, ethnicities, socioeconomic status, and age [7,14]. Despite the existence of digital health platforms, some subgroups may lack awareness of their availability and knowledge in usage. The exorbitant costs of devices and unstable connectivity render digital health a luxury for these individuals. To aggravate this issue, poor health outcomes requiring urgent needs for quality healthcare, are associated with lower standards of living or income brackets [7,14].

**Fig 1:**
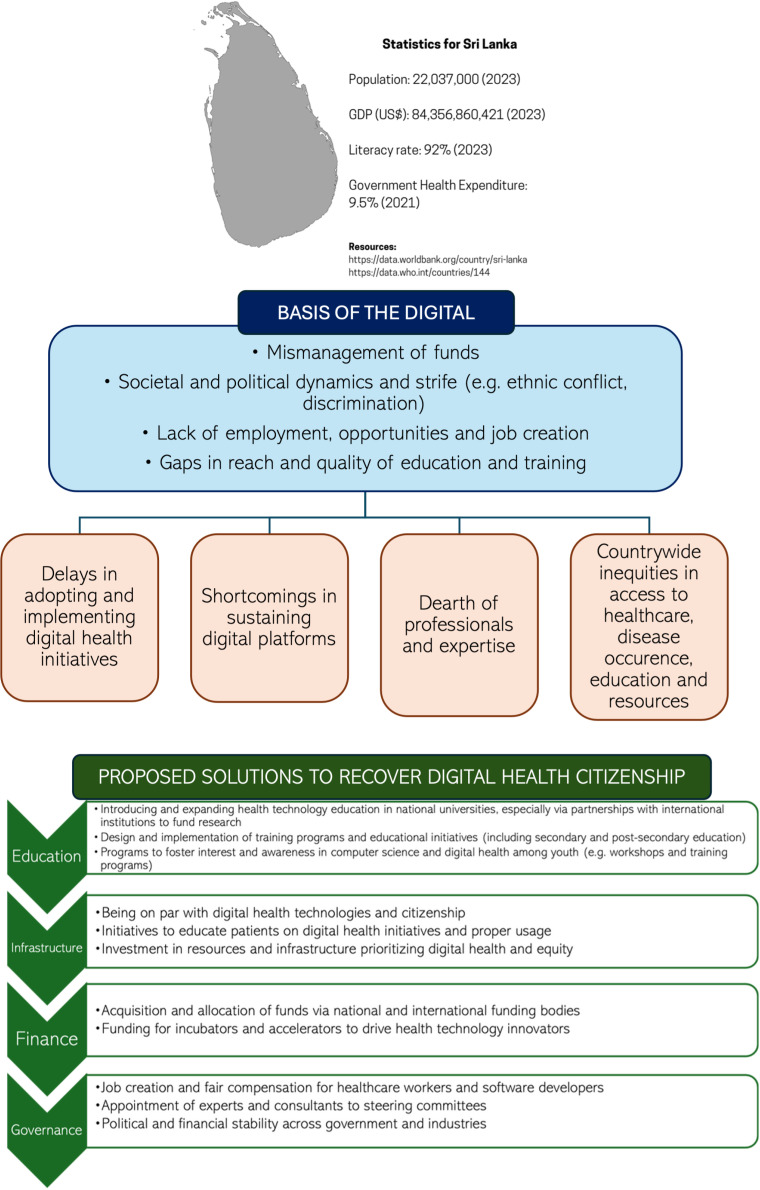
Summary of digital health citizenship in Sri Lanka in the context of (1) its past and current position, (2) the economic crisis, and (3) proposed strategies to overcome key setbacks [ 15].

These are only some of the innumerable challenges threatening digital health citizenship in Sri Lanka. Digital health citizenship during the economic crisis presents a fresh challenge for Sri Lanka’s healthcare systems. As summarized in Fig 1, the Government of Sri Lanka must mobilize strategies to foster youth engagement in digital health citizenship, primarily through awareness, opportunities and sustainment of these initiatives and digital health platforms [1].

## Abbreviations:

LMICs: low- and middle-income countries
SVC: Smart Vaccination Certificate
COVID-19: coronavirus disease
STD/AIDS: sexually transmitted disease/acquired immunodeficiency syndrome
HIV: human immunodeficiency virus
